# Glycans as Potential Diagnostic Markers of Traumatic Brain Injury in Children

**DOI:** 10.3390/diagnostics13132181

**Published:** 2023-06-26

**Authors:** Mårten Kvist, Lasse Välimaa, Adrian Harel, Sari Malmi, Aleksi Tuomisto

**Affiliations:** 1 Medicortex Finland Plc, 20520 Turku, Finland; 2 Department of Pediatric Surgery, Satasairaala Hospital, 28500 Pori, Finland

**Keywords:** TBI, lectin, glycan, biomarker, concussion, traumatic brain injury, clinical trial, diagnostic test, children

## Abstract

Diagnosing mild traumatic brain injury (TBI) in the acute setting is challenging due to the nonspecific and often transient or delayed symptoms. Further, the criteria for acute head imaging are frequently not fulfilled, which may lead to a missed diagnosis. A rapid test to diagnose TBI using body fluids would be highly useful. Urine and saliva samples were collected from 28 pediatric patients (mean [SD] age, eight years two months [four years three months]) with acute, clinically diagnosed mild TBI and 30 healthy volunteers at Satasairaala Hospital, Pori, Finland, over 11 months. The mean (SD) time from trauma to first sampling was 3 h 56 min (1 h 14 min). Samples were analyzed to determine the number of lectin-binding glycan molecules, indicating nerve tissue damage. The relative levels of several lectin-bound glycans were measured by fluorescence. Compared with healthy controls, the TBI group showed significant increases (*p* < 0.05, Wilcoxon rank-sum two-sided test) in nine glycans in the saliva, one glycan in the urine, and a significant decrease in seven glycans in the urine. These findings of potentially diagnostic glycans in body fluids after TBI warrant further research and may enable the development of a rapid body fluid-based point-of-care test to identify pediatric patients with TBI after a head injury.

## 1. Introduction

An impact to the head or rapid movement of the head, may cause a traumatic brain injury (TBI), which leads to altered brain function. Mild traumatic brain injury (mTBI), especially its mildest form, concussion, is particularly challenging to diagnose [1,2,3].

Computed tomography (CT) is used to detect traumatic intracranial findings in patients with head injuries, but it cannot be used to exclude TBI. In addition to CT, some biomarkers may detect brain injuries, e.g., S100β, which was introduced more than 50 years ago [4]. Recently, the use of S100β as a biomarker has been recommended in Scandinavian countries, and it has been validated in the Finnish population [5]. A recent study assessing whether S-100β protein could be measured in the urine after its detection in the plasma following mTBI, however, found that urine sampling for S-100β protein was not useful during the acute phase [6]. To detect intracranial injuries, the US Food and Drug Administration has approved glial fibrillary acidic protein and ubiquitin carboxy-terminal hydrolase L1 (GFAP-UCH-L1) [7]. Some biomarkers for detecting mTBI have been developed over the years, but none are considered ideal or have the potential to replace clinical evaluation and CT. Review articles of biomarkers available for TBI have been published by Carney et al., Harel et al., and Wang et al. [8,9,10].

Alterations in glycans have been shown to indicate TBI in adult patients [11]. The present study was conducted to collect clinical samples from children for the discovery of a novel biomarker for TBI. Using either saliva or urine instead of blood for testing is a potential advantage in clinical practice. The intention of this study was to demonstrate the possibility of using saliva or urine as a source of biomarkers for detecting mTBI. A rapid diagnostic test based on a new biomarker would significantly improve the quality of the diagnostic process concerning mTBI. This will improve the treatment of patients with head injuries [9,12,13].

Our hypothesis was that altered levels of some glycans may function as early indicators of brain injury in children.

## 2. Materials and Methods

### 2.1. Study Design

This was a small prospective study of children with fresh mTBI. Their saliva and urine samples were compared with those of uninjured, healthy controls. Possible differences in the glycan concentrations and compositions were analyzed using lectin-binding methods. The study was approved by The Ethics Committee of the Hospital District of Southwest Finland on 17 December 2019 (T257/2019). An amendment to the protocol was approved on 4 August 2020. The study was registered on 28 February 2020 in clinical trials with the registration number NCT04288167. The clinical phase of the study—registration of the patients and collection of the body fluid samples—was conducted between 14 May 2020 and 13 April 2021.

### 2.2. Settings and Population

A total of 28 patients who had suffered an impact to the head and had been hospitalized for suspected TBI at Satasairaala in Pori, Finland, were recruited. All patients were under 18 years of age. After obtaining informed consent from the patients/parents/guardian to participate in the study, body fluid samples were collected. All 28 patients with suspected TBI were diagnosed with mTBI.

The patients, who were treated in the Emergency Department of the hospital because of a clinically diagnosed mTBI, were classified as TBI patients (total of 28 patients). The control group comprised 30 uninjured volunteers who had not suffered any head trauma during the previous three months. The diagnosis of positive TBI required a pediatrician’s assessment of the case history, injury mechanism, and symptoms in combination with the pediatric Glasgow Coma Score (GCS) evaluation and brain imaging results. Patients with relevant TBI-related symptoms and a GCS of 13–15 were classified as having mTBI.

From the patients with suspected TBI, 1–2 mL of saliva and ~10 mL of urine were collected at two time points after the injury. Saliva samples were collected at least 1 h after eating. In children aged ≤4 years, saliva samples were collected using a syringe without a needle from the sublingual space in the mouth. Patients >4 years of age were asked to rinse their mouths twice with pure water and then to spit saliva into a clean plastic cup. Urine samples were collected in a plastic cup or potty and transferred to storage vials.

All specimens were frozen as quickly as possible at a temperature of −70 °C and stored (up to a few months) until analyzed together in the laboratory.

Existing international guidelines were followed in the treatment of all patients in the study [2].

Samples from healthy controls, comprising mainly children of staff members of Satasairaala, were obtained as indicated and scheduled between the subject or guardian and the study coordinator. Thirty subjects who had not suffered any head trauma (self-reported) in the past three months were included as healthy controls, and their urine and saliva samples were taken once using similar methods as for the samples of the injured patients.

### 2.3. Inclusion Criteria

The inclusion criteria were:

1. Presenting to the hospital with suspected TBI (isolated) occurring no more than 6 h before arrival at the hospital (with the first sampling time occurring at a maximum of 6 h after the injury);
2. GCS ranging from 13 to 15 (mild TBI or concussion);
3. Patient conscious at the time of recruitment;
4. Signed written informed consent form, in Finnish or Swedish, signed by the parent/legal guardian and also by the study subject if he/she was considered capable of understanding the study measures;
5. Age 0–17 years (male or female).

### 2.4. Exclusion Criteria

The exclusion criteria were:

1. Unknown time of trauma (uncertainty >1 h).
2. More than 6 h elapsed since the injury incident.
3. Multi-trauma patient, or history of head injury, seizures, or stroke within 3 months of the current presentation at the emergency department.
4. Patient suspected to be drunk or intoxicated due to alcohol or drug use.
5. Current use of anti-psychotic, anxiolytic, or antiepileptic medication.
6. Known pre-existing neurologic conditions that can cause the observed symptoms.
7. Chronic neurodegenerative, metabolic (e.g., diabetes), or autoimmune disease.
8. Suspicion of pregnancy.
9. History of HIV or hepatitis B virus infection.

The final diagnosis was assigned to the patient when they left the hospital. The diagnosis was based on the overall clinical examination, measurement of the GCS, and the radiologist’s statement of the possible findings in a CT scan. All patients in the TBI group had commotion as their final diagnosis.

During the study period, one remote monitoring event (video connection) in the middle of the study and a site visit at the closure were made by an external clinical study monitor to ensure that data were collected and recorded according to the protocol.

### 2.5. Biochemical Analysis Procedures

Glycans bind to lectins, which are mainly plant-based proteins that bind carbohydrates with high specificity for their target glycan structure and a moderate binding affinity. The samples were analyzed by an ISO-certified contract laboratory (Tebu-Bio Ltd., Le Perray-en-Yvelines, France) to screen for lectin-binding glycans present in the samples. The laboratory had no information about the division of the samples into the study groups. The biochemical measurements were thus performed blinded.

A biochemical glycan-binding analysis was used for the sample analysis [14]. Each sample was applied to a glass slide containing 95 different lectins printed on the surface to capture the glycans of interest from the sample. The visualization of bound glycans was enabled using a fluorescent reporter complex. The lectins used for screening represented multiple different glycan binding specificities (Lectin Array 95, RayBiotech^®^, Peachtree Corners, GA, USA). The fluorescence intensity measured from each lectin spot was proportional to the amount of that lectin-binding glycan in the sample. The levels and intensities were analyzed and compared between the study groups.

Statistical analyses of the results were conducted using a t-test for comparisons between the TBI group and the healthy control subjects. *p*-values less than 0.05 were considered statistically significant, and the fold-change was calculated to indicate the relationship of the mean values between the TBI group and controls.

## 3. Results

The age and sex distributions of the TBI patients and healthy controls are presented in Table 1.

Healthy controls were age- and sex-matched with TBI patients.

The mean age in the TBI group was eight years two months (SD four years three months, median seven years two months), and that in the control group was seven years eleven months (SD 4 years six months, median seven years five months).

### 3.1. TBI Trauma Mechanisms

Among the 28 TBI patients, the TBI was caused by a fall in 18, a blow to the head in 4, a motor vehicle accident in 4, and a kick to the head in 2. A bicycle was involved in 5 accidents, 4 patients were injured in sports activities, and 5 were injured while playing. Although the site of the accident was not requested to be recorded in the study database, it can be deduced from the injury mechanism descriptions that many types of accidents were represented, including home accidents, school accidents, playground accidents, sports accidents, and traffic accidents. Additionally, the use of helmets at the time of the accident was not recorded; 1 of the bicyclists was not wearing a helmet, while another was wearing a helmet at the time of injury.

### 3.2. Time Elapsed between Injury and Testing

For the injured, the mean (SD) time elapsed since the injury and the first sample collection was 4 h 21 min (1 h 10 min; median 3 h 32 min; minimum 1 h 4 min; maximum 6 h 25 min). The mean (SD) time between injury and arrival at the hospital was 1 h 46 min (1 h 23 min). The shortest amount of time between the injury and testing was 36 min and the longest time was 5 h 56 min.

The first saliva and urine samples from patients with suspected TBI were collected a mean (SD) of 1 h 52 min (1 h 5 min) after arrival at the hospital (median 1 h 41 min, minimum 16 min, and maximum 4 h 17 min).

For one patient, the first sample was obtained 6 h 40 min after the injury, which, strictly speaking, was not in accordance with the protocol (maximum allowed time 6 h after injury), but it was included as the time overrun was considered minor.

The second sampling was supposed to be performed at least 4 h after the first sampling but before the patient was discharged from the hospital. The second sampling was performed at a mean (SD) of 11 h 04 min (4 h 35 min; median 9 h 38 min; minimum 5 h 1 min; maximum 21 h 55 min) after the injury. The protocol suggested collecting the second sample within 10 h after the injury, but this was challenging for various practical reasons, and 14 urine samples and 15 saliva samples were obtained later than indicated in the protocol. Table 2 shows how the samples were collected over time after the TBI.

### 3.3. Clinical Findings

Of the 28 patients treated in the hospital for TBI, all were eventually diagnosed with mTBI. The patients were treated according to existing guidelines [2].

Five patients had become unconscious immediately after the accident, with the duration of the unconsciousness in all cases being <5 min. At the time of recruitment, all patients were conscious. Post-traumatic amnesia was detected in six patients, and the longest duration of amnesia was 2 h.

The GCS were initially assessed as being between 13 and 15. For 16 of the 28 patients, the GCS remained unchanged at 15 during the entire hospital stay.

For all TBI patients, the trauma codes are listed in Table 3, and the concomitant chronic disease codes for the whole study group are shown in Table 4.

### 3.4. Vital Signs

The mean (SD) systolic blood pressure upon arrival at the hospital was 111 mm Hg (13.4 mm Hg), and the mean diastolic pressure was 68 mm Hg (12.0 mm Hg). The mean oxygen saturation percentage was 99.1 (1.21). The mean pulse rate was 90.4/min (21.0/min). The mean respiration rate at admission measured for 20 patients was 19.1/min (4.30/min).

Among the 28 patients, blood sugar was measured for 15 patients, and the mean blood sugar level was 6.8 mmol/L (2.06 mmol/L).

The GCS was measured twice: upon arrival at the hospital and again 4 to 10 h later. The lowest GCS value recorded was 13 in 1 patient, and 6 patients had GCS 14, whereas all the others had GCS 15.

### 3.5. Symptoms and Signs

The symptoms and signs presented by the pediatric patients upon arrival at the Emergency Department are listed in Table 5. The symptoms are listed according to a predefined list of possible symptoms or signs. The staff was able to add other symptoms that were not prelisted. Headache and nausea were the most common symptoms, present in more than half of the TBI patients, whereas other symptoms varied. Dizziness and crying were reported in more than a third of the cases. The other most common symptom was tiredness, but visual defects and thumb pain were also reported. All patients had at least one of the prelisted symptoms.

### 3.6. Cognitive Symptoms

Cognitive disturbances were recorded according to a predefined list of the ten most anticipated cognitive defects after a TBI. The results are shown in Table 5. The most common disturbances were disorientation and slowness of thinking, which occurred in half of the patients. Slow information handling and an impaired sense of space were also common. At most, one patient had five listed cognitive disturbances, whereas ten patients had no cognitive disturbances.

### 3.7. Imaging Results

A CT scan was performed on nine patients. Additionally, later magnetic resonance imaging (MRI) studies were performed in eight patients, seven of whom had also undergone CT imaging. In only one case did the MRI show additional remarkable findings not detected by the previous CT scan. The CT or MRI scan was regarded by the clinician as indicative of a TBI only in three patients. The statements of the radiologist revealed no mention of signs of TBI in the images in two of these cases. One patient had a mandibular fracture; another had a suspected fissure in the occipital region; and the third patient had a small single hemosiderin plot in the cerebellar region, indicative of a possible TBI. According to the radiologist, the etiology in this case could not be assessed with certainty.

### 3.8. Biomarker Results

The results of the measurements of the glycans in the saliva and urine are shown in Table 6. Only results with significant differences are shown.

All glycans listed in the table exhibited statistically significant (*p* < 0.05) differences between TBI patients and controls.

**Figure 1:**
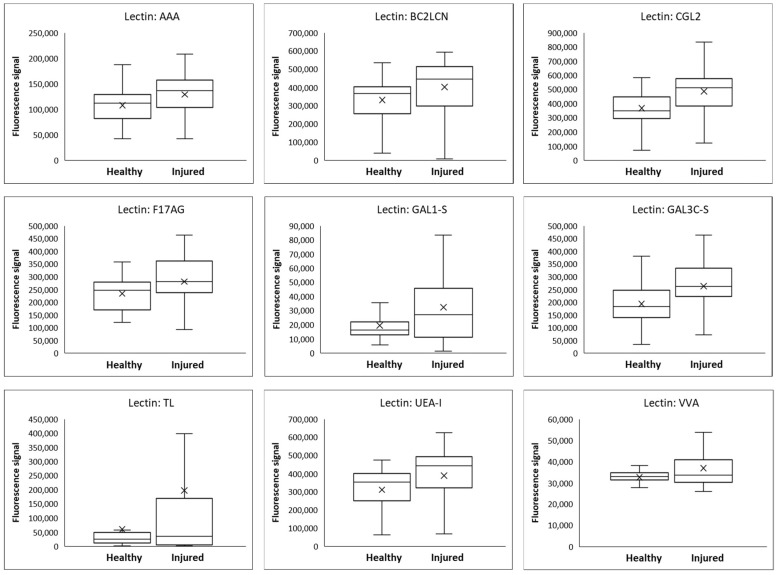
Box plots showing lectin-binding signal levels and distributions in saliva for nine significant lectins in samples from patients with mild TBI (28 patients, two samples from each) in comparison to healthy control subjects (30 subjects, one sample from each). Interpretation of the box plots: whisker low end: minimum value; lower edge of the box: 1st quartile; tick: average value; horizontal line inside the box: median; upper edge of the box: 3rd quartile; whisker top: maximum value.

**Figure 2:**
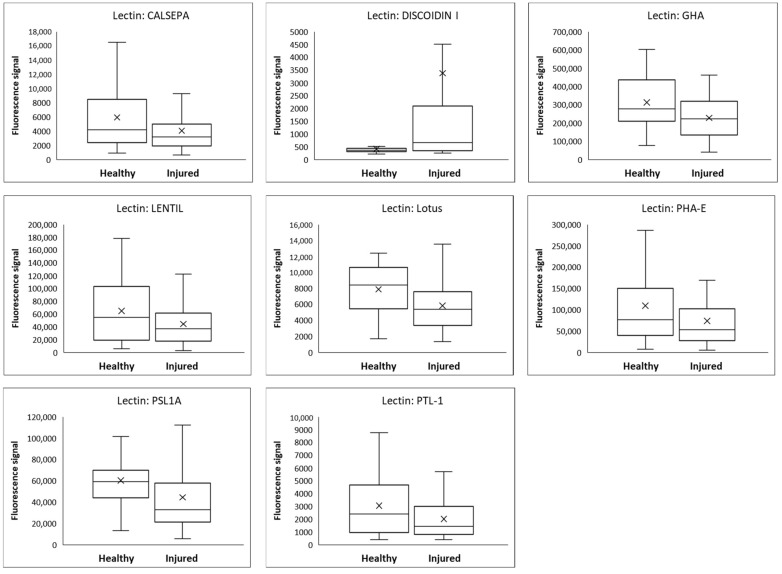
Lectin-binding signal levels and distributions in urine for eight significant lectins in samples from patients with mild TBI (28 patients, two samples from each) in comparison to healthy control subjects (30 subjects, one sample from each). Interpretation of the box plots: whisker low end: minimum value; lower edge of the box: 1st quartile; tick: average value; horizontal line inside the box: median; upper edge of the box: 3rd quartile; whisker top: maximum value.

Compared with healthy controls, all patients had statistically significant differences in at least some glycans in both saliva and urine. In saliva, nine glycans showed a significantly increased values in TBI patients and none showed a decreased value, whereas one glycan in the urine showed significantly increased value and seven glycans showed a decreased value in TBI patients (Table 6). Box-plot diagrams for saliva are shown in Figure 1 and for urine in Figure 2.

### 3.9. Glycan Analysis Results over Time

As two samples over time were obtained from the patients with TBI, it was possible to deduce how the biomarker results might change over time. For analysis, the time frame for the recruitment of specimens was divided into three groups: (1) <6 h, (2) 6–10 h, and (3) >10 h after the injury (Table 2). The mean difference between the first and second samplings was 7 h 21 min.

In saliva, the lectin-binding glycan results were almost unchanged between the first and second time periods, whereas in the third time period, only 16% showed increased values and 84% showed decreased values compared with the initial test results.

In urine, most of the lectin-binding glycans (94/95) showed a higher concentration in the second and third periods compared with the initial test results. About 22% of the lectin-binding glycan values increased continuously from the initial measurement to the last.

Among the 28 TBI patients, the saliva samples showed decreased values in the second sample in 50% of the patients, whereas the urine samples showed decreased values in the second sample in only 21% of the patients. Increased values were recorded in saliva samples in 39% of the cases, and an increase was detected in the urine in 43% of the cases.

### 3.10. Correlation between Clinical Findings, Cognitive Symptoms, and Glycan Analysis Results

Of the ten TBI patients who had imaging performed (either a CT scan or MRI of the head) after the TBI, three were considered by the treating clinician to have a possible pathologic finding.

One patient, a 15-year-old boy with an undisputable TBI finding on MRI, had increased values for eight lectin-binding values in saliva (LPA, WFA, DISCOIDIN-II, RCA 120, SBA, LEA, PA-IL, and STL), whereas half of the tested lectins showed decreased values in comparison with the healthy controls. A significant difference was considered that exceeding or below the healthy control values by two SD. Clinically, this patient, who had not been unconscious, was suffering from headaches, dizziness, slowness of thinking and information handling, disorientation, and amnesia. The GCS was 14. The other vital signs (blood pressure, SaO_2_, blood sugar, pulse rate, and respiration rate) were normal. The same patient had slightly higher values for some glycans in the urine sample, but the values were not statistically significant.

Among the TBI patients, a patient with a mandibular fracture had very high values of the glycan binding to the lectin DISCOIDIN (second highest value among all study subjects) in the urine. Among the other patients with a significant (>2 SD of the healthy subjects) increase of this glycan in the urine, the health status of seven patients did not initially indicate the necessity for a CT scan or MRI.

One of the lectins (DISCOIDIN) showed an eight-fold mean increase in the TBI patients compared with the healthy controls.

Of the patients showing no signs of TBI at imaging, five had a GCS score of 14.

The patients with a more severe trauma history among the mild TBI cases had more somatic symptoms such as headache, crying, dizziness, nausea, and vomiting. The cognitive symptoms (e.g., disorientation, slowness of thinking, impaired sense of space, and slow information handling) correlated well with the somatic symptoms. All the patients with recorded cognitive disturbances (*n* = 18) had statistically significant glycan results in at least three of the four samples (two saliva + two urine) taken at two sampling events.

Among the healthy controls, two study subjects showed significant changes in glycan levels in saliva, and two others showed significant changes in glycan levels in urine; neither were aware of any previous TBI.

## 4. Discussion

Glycans are components of glycoproteins and other glycosylated macromolecules that are released from nerve tissue in the brain when a patient incurs a TBI and damage to the blood-brain barrier. The amounts of glycans can be measured in vitro by lectin-based bio affinity binding assays. Lectins are carbohydrate-binding proteins originating mainly from plants. Different lectins have different affinities for different glycan structures or sequences.

The null hypothesis for this study was that no differences in the glycan concentrations of the two tested body fluid types (saliva and urine) would be detected in pediatric patients with acute TBI in comparison with healthy subjects. Analysis of the glycan content in body fluids revealed significant changes in both types of body fluid. Significant increases in nine lectins, indicating eight different glycans in saliva and one in urine, were detected in suspected TBI patients. Thus, the null hypothesis could be rejected.

The diagnosis of mTBI is troublesome and controversial [1,2]. It is also controversial whether mTBI can be reflected by abnormal CT scan results. According to the current guidelines for the management of TBI, a small abnormal finding in the imaging may still be classified as mTBI when the duration of the loss of consciousness and posttraumatic amnesia is short [1,15]. Even if the GCS is 13–15, a positive finding from a CT scan is always evidence of mTBI [16].

In the present study, the diagnosis of TBI was made by the clinician in charge of the patients in the Emergency Department. The diagnosis was based on both clinical evidence, the GCS score, and CT imaging results. However, the information may still be inconclusive. Additional diagnostic means, such as biomarkers, may fill the current gap in mTBI diagnostics.

A few biomarkers show promise for detecting TBI [5,7,8]. Currently, S100β is the only biomarker recommended by task groups such as the Scandinavian Guidelines for the diagnosis of head injuries [2]. Other injuries may also influence the level of this biomarker, however, making it less specific [17]. In 2018, the US Food and Drug Administration authorized the first blood test to aid in the evaluation of concussions in adults [18].

Most of the biomarkers studied focus on measuring proteins in the blood or plasma. Here, we focused on changes in glycan components in the saliva and urine obtained from patients with acute TBI. The aim was to discover brain trauma indicators that could be measured by non-invasive sampling and, owing to easy sampling, also be applicable for use in an ambulatory setting. This study tentatively indicated altered responses of certain glycans both in saliva and in urine in patients with TBI compared to healthy subjects. The biggest differences between TBI samples and control samples were found in the urine (Table 6).

The use of a valid and reliable biomarker would be a valuable addition to the diagnostic process for TBI in conjunction with clinical evaluation and imaging diagnostics. The hazards of radiation exposure are well known, as reported by Brenner and Hall, Smith-Bindman et al., and Braganza et al. [19,20,21].

In this study, the patient group was selected among children treated in a hospital ward for suspected acute TBI. At hospital discharge, all the patients had received a diagnosis of mTBI. Family members of the hospital staff members formed the healthy control group. Surprisingly, among the healthy controls, two study subjects showed significant changes in glycan levels in saliva, and two others showed significant changes in glycan levels in urine; neither were aware of any previous TBI. It is possible that the subjects had earlier suffered a mTBI but were not aware of the symptoms at the time of recruitment into the study, thereby considering themselves healthy.

Males are generally overrepresented among TBI patients in comparison to females [22]. In order to secure age and sex matching in each study group, the recruitment period was extended in this study until adequate matching was obtained.

Of the 28 patients hospitalized with TBI, only three (11%) were assessed to have TBI-induced changes in brain imaging (either CT or MRI). In other studies, the prevalence of positive findings varied between 4.7% and 19% among TBI patients that underwent brain CT imaging [8]. The number of patients in this study was limited, and thus it is premature to conclude whether glycan detection could help with decision-making in cases of mTBI. The main objective of the study was to determine whether concentrations of glycans were altered by brain injury. The findings are of sufficient interest to continue with the study by including more patients with only mTBI and CT or MRI images showing clear traumatic lesions.

Significant increases in some glycans were observed in the saliva and urine, implying that, upon further development, such glycans may serve as biomarkers of TBI and could possibly be detected with a rapid and portable diagnostic kit. The use of a rapid test to detect TBI offers several advantages [3]. A rapid test would facilitate decision-making when the test results are easily available at a low cost. A sufficiently sensitive biomarker test may accelerate and facilitate the diagnostic process and thus reduce the need for a head CT scan. A biomarker test can easily be repeated when the initial results are negative but suspicion of mTBI remains. A multivariate analysis of all 95 lectin-binding results could potentially offer additional information on usable combinations of biomarkers for testing TBI.

One patient with TBI had a surprisingly pathologically elevated blood sugar level (11.2 mmol/L), implying diabetes. Strictly speaking, the patient should have been excluded from the study based on the exclusion criteria but was included as the disease was not previously diagnosed. The mean (SD) blood sugar level for the injured patients was 6.8 mmol/L (2.06 mmol/L).

The accuracy of the assessment depends on the number of people making the patient assessments. In this study, the GCS scores were independently assessed by a total of 15 health professionals; radiologic statements were made by eight radiologists; and the number of doctors in charge of the treatment was eight. The fact that multiple people participated in the treatment of the patients may lead to some variation among the results, but this is difficult to control and normalize in a clinical setting.

Imaging, while considered the gold standard for assessing brain damage, carries a risk of radiation. In general, CT is available in the emergency department, whereas MRI is not. Children’s brains are extremely vulnerable to radiation [19,20,21,22] and a CT of the brain introduces a radiation dose of 2.1 mSv [19]. In the present study, only 10 of 28 patients underwent a head scan by either CT or MRI. In cases of mTBI, radiologic reports have limited use for assessing TBI cases. Patients suspected of having a more severe injury were referred for imaging. The clinicians found three patients with signs of brain injury based on the imaging, but according to the statements of the radiologists, only one patient had possible intracerebral bleeding. The other two patients had skull bone fractures, but no brain bleeding. The results are consistent with expectations, as the selection criterion for the study group was suspected mild TBI.

The glycan results are parallel to the clinical assessment since all patients had statistically significant differences in at least some glycans in both saliva and urine in comparison to the healthy group. It may be assumed that glycans, or something that affects glycan detection, are released by a rupture of the blood-brain barrier, and altered glycan levels serve as an indication of brain injury. Intact brain blood barrier levels would be associated with normal glycan levels.

The literature on the connection between human TBI and glycans is limited. Glycosylation is a common post-translational modification, and it is suggested that more than half of all proteins are glycosylated [23]. The concept of glycans as potential biomarkers of disease has only recently emerged. Changes in glycan expression in cancer cells have been studied as a cancer diagnostic, and recent research on glycan biomarkers has yielded several new diagnostic approaches [24,25]. For example, a glycosylated fraction of α-fetoprotein (AFP), a marker for liver tumor, was found to discriminate hepatocellular carcinoma from benign liver disease better than the total protein analysis of AFP [26,27]. In the central nervous system, changes in glycan profiles have been detected in animal studies in connection with neurological pathologies and the development of neurodegenerative conditions after TBI [28,29]. A recent study reported alterations in human serum glycome in moderate and severe TBI [30].

The clinical usefulness of biomarkers for mTBI diagnostics is overt, as the results of the study suggest. The use of a biomarker test does not require the involvement of professional staff nor sophisticated imaging equipment. It will be time-saving and expedite the diagnostic process. The ideal biomarker of brain injury should exclude a brain injury and thereby reduce the need for acute radiological examinations. This study involved a small number of patients, and therefore, all findings should be regarded as preliminary. However, glycans may bear potential for serving as biomarkers of brain injury, and they can be detected using straightforward binding tests, which grounds the development of easier and more affordable detection methods for concussion and mild brain injury.

## 5. Conclusions

This study had sufficient samples from both TBI patients and healthy controls to proceed with the development of biomarkers for rapid diagnosis of TBI suitable for use in children. As a proof-of-concept, significant differences in the concentrations of nine glycans in saliva and eight in urine were found between TBI patients and healthy controls.

## Figures and Tables

**Table 1:** Distribution of the study subjects according to age group and sex.

Age Group (Yrs.)	TBI Patients			Healthy Controls			All
	Males	Females	Total	Males	Females	Total	Total
0–4	5	3	8	5	5	10	18
5–9	5	5	10	5	5	10	20
10–17	5	5	10	5	5	10	20
Total	15	13	28	15	15	30	58
Age range (y)			1–15			0–17	

TBI, traumatic brain injury.

**Table 2:** Distribution of sample collection over time.

	Time Window (from Injury to Sampling)		
	<6 h	6–10 h	>10 h
**Urine**			
1st sample (*n*)	27	1	-
Mean time (h:min)	3:50	6:40	*-*
SD time	1:08	-	*-*
Median time	3:40	-	-
2nd sample (*n*)	1	13	14
Mean time (h:min)	5:01	6:51	14:58
SD time	-	3:04	4:00
Median time	-	7:40	14:04
**Saliva**			
1st sample (*n*)	27	1	-
Mean time (h:min)	3:45	6:25	-
SD time	0:58	-	-
Median time	3:38	-	-
2nd sample (*n*)	1	12	15
Mean time (h:min)	5:00	7:31	15:17
SD time	-	1:09	3:32
Median time	-	7:27	14:15

SD: Standard deviation.

**Table 3:** Trauma diagnosis codes of the patients with suspected TBI.

Main Diagnosis (ICD-10 Code)	Type of Trauma	*n*
S06.0	Concussion	28
S01.1	Lid or periorbital wound	1
S01.3	Ear wound	1
S01.8	Head wound, other	1
S02.69	Fracture of the jaw	1
S02.8	Other fracture of the face, closed	1
S02.9	Other fracture of the skull or face	1
S40.0	Contusion of the shoulder	1

ICD-10: International Classification of Diseases, version 10.

**Table 4:** Concomitant diseases among the study subjects.

ICD-10 Code	Group	*n*	Comorbidities
F50.0	Control	2	Asthenia
J45.0	Control	2	Allergic asthma
K21.9	Control	2	Gastro-esophageal reflux disease
K91.2	Control	1	Postoperative malabsorption
Q21.2	Control	1	Atrio-ventricular defects
Q25.5	Control	1	Open ductus botalli
Q43.1	Control	2	Hirschsprung’s disease
Q07.0	TBI	1	Arnold-Chiari syndrome
Q87.27	TBI	1	CHIARI-association

ICD-10: International Classification of Diseases, version 10.

**Table 5:** Somatic and cognitive symptoms of TBI patients presented upon hospital arrival.

Somatic Symptoms	*n*	%
Headache	17	61
Nausea	16	57
Crying	12	43
Dizziness	12	43
Vomiting	9	32
Bleeding	3	11
Tiredness	3	11
Neck stiffness	2	7
Apnea	1	4
Convulsions	1	4
Coma	1	4
Tremor	1	4
Visual defects	1	4
**Cognitive symptoms**		
Disorientation	14	50
Slowness of thinking	14	50
Impaired sense of space	11	39
Slow information handling	11	39
Irritability	3	11
Disorder of attention	2	7
Emotional instability	1	4

**Table 6:** Fluorescence measurement intensities in the saliva and urine of 58 pediatric study subjects grouped as patients with TBI and uninjured healthy controls.

Lectin	TBI		Healthy			
	Mean	SD	Mean	SD	TBI versus Healthy	*t*-Test *p*-Value
Saliva:						
AAA	128,944	40,902	107,981	36,104	1.19	0.022
BC2LCN	401,948	143,196	330,504	121,254	1.22	0.024
CGL2	486,078	199,934	367,029	130,596	1.32	0.005
F17AG	280,365	107,380	233,060	67,557	1.20	0.033
GAL1-S	32,401	25,892	19,408	9870	1.67	0.011
GAL3C-S	262,447	105,055	192,852	80,462	1.36	0.002
TL	197,503	344,531	59,823	87,434	3.30	0.036
UEA-I	388,619	156,320	310,849	126,795	1.25	0.023
VVA	36,907	8903	32,619	2810	1.13	0.013
Urine:						
CALSEPA	4057	3089	5916	4919	0.69	0.037
DISCOIDIN I	3373	5702	410	177	8.22	0.006
GHA	228,238	116,064	313,121	138,179	0.73	0.004
LENTIL	44,274	37,160	64,776	50,186	0.68	0.037
Lotus	5842	2937	7893	3005	0.74	0.003
PHA-E	73,493	60,407	109,517	93,657	0.67	0.036
PSL1A	44,469	34,794	60,245	29,779	0.74	0.041
PTL-1	2002	1579	3058	2531	0.65	0.021

## Data Availability

Original patient data stored at the Department of Pediatric Surgery at Satasairaala Pori, Finland. Laboratory data stored at Medicortex Finland Plc, Itäinen Pitkäkatu 4 B, 20520 Turku, Finland. Documentation of the clinical trial: clinicaltrials.gov, Identifier NCT04288167, https://clinicaltrials.gov/ct2/show/NCT04288167?term=medicortex&draw=2&rank=2 (accessed on 24 January 2023).

## Abbreviations

CT: computed tomography; MRI: magnetic resonance imaging; mTBI: mild traumatic brain injury; TBI: traumatic brain injury.
